# Effects of L-Arginine on Bone Metabolism: Evidence from In Vitro and In Vivo Models

**DOI:** 10.3390/ijms26178484

**Published:** 2025-09-01

**Authors:** Clara Pertusa, Álvaro Carrasco-García, Rosa Aliaga, Loreto Suay, Eulalia Alonso-Iglesias, Antonio Cano, Juan J. Tarín, Miguel Ángel García-Pérez

**Affiliations:** 1 Research Unit, INCLIVA Biomedical Research Institute, 46010 Valencia, Spain; clara.pertusa@uv.es; 2 Department of Internal Medicine, Erasmus MC, 3015 CE Rotterdam, The Netherlands; al.carrasco.garcia@gmail.com; 3 Department of Paediatrics, Obstetrics and Gynaecology, University of Valencia, 46010 Valencia, Spain; rosa.m.aliaga@uv.es (R.A.); m.loreto.suay@uv.es (L.S.); 4 Department of Biochemistry and Molecular Biology, University of Valencia, 46010 Valencia, Spain; eulalia.alonso@uv.es; 5 Salus Vitae Women’s Health Clinical Centre, 46004 Valencia, Spain; antonio.cano@uv.es; 6 Department of Cellular Biology, Functional Biology and Physical Anthropology, University of Valencia, 46100 Burjassot, Spain; juan.j.tarin@uv.es; 7 Department of Genetics, University of Valencia, 46100 Burjassot, Spain

**Keywords:** L-arginine, ovariectomy, mice, Saos-2, bone microarchitecture

## Abstract

Despite the rising incidence of osteoporosis (the most common bone disorder) as life expectancy increases worldwide, the genetic and metabolic factors contributing to this multifactorial disease are still poorly understood. This study investigated the role of arginine metabolism in bone formation and its potential for preventing bone loss in postmenopausal osteoporosis. The osteogenic effects of arginine were evaluated in vitro by determining calcium mineral deposition and the expression of marker genes in the human osteoblastic cell line Saos-2. In vivo analyses were conducted in ovariectomized mice treated with arginine, focusing on femoral bone microarchitecture, marker gene expression and serum metabolite profiles. Arginine treatment enhanced calcium deposition and osteoblastic differentiation in vitro. In contrast, however, this treatment had a deleterious effect in vivo, exacerbating trabecular bone loss. These results are particularly relevant given the wide availability of arginine as a dietary supplement, and our findings underscore the necessity of verifying the safety of nutritional supplements in different populations and in the presence of disease.

## 1. Introduction

Osteoporosis (OTP) is a systemic skeletal disease characterized by low bone mass and deterioration of the bone microarchitecture, which diminishes bone resistance and significantly predisposes individuals to fragility bone fracture. Postmenopausal OTP is caused by decreased estrogen production by the ovaries, leading to an imbalance in bone remodeling whereby bone resorption outpaces bone formation. The resulting abrupt bone loss contributes to a greater incidence in women of OTP, the most prevalent musculoskeletal disease in the female elderly western population with sequelae severely affecting patient functional status [1].

OTP is diagnosed by measuring bone mineral density (BMD), for which the most accurate currently available method is dual-energy X-ray absorptiometry (DXA) [2]. OTP is a multifactorial disease, and BMD is influenced by genetic, epigenetic, and environmental components [3]. The research aims of our group include identifying novel genes and polymorphisms associated with BMD through a translational approach using ovariectomized (OVX) mice as model of accelerated bone loss. In a previous study, we identified two genes involved in arginine metabolism, glycine amidinotransferase (*Gatm*) and spermine oxidase (*Smox*), whose expression was significantly reduced in the bone marrow B-cells of OVX mice [4].

L-arginine (Arg) is a semi-essential amino acid whose role in bone metabolism and bone fracture has been widely debated due to its involvement in nitric oxide (NO) production and in the synthesis of polyamines, among others. Arginine has been found to increase type I collagen synthesis in primary osteoblasts isolated from osteopenic rats [5] and to have a protective effect against bone resorption induced by administrating zinc oxide nanoparticles in the same species [6]. In postmenopausal women, however, arginine administration did not reverse bone loss [7].

This background, including our own results in animal models and the inconclusive data in the literature on the role of arginine in bone metabolism, forms the rationale for the present study. Our main objectives are twofold: first, to analyze the osteogenic capacity of arginine in the human osteoblastic cell line Saos-2, and second to establish whether arginine administration prevents BMD loss in the ovariectomized mouse model. These aims are particularly relevant given that L-arginine supplements are currently administered without prescription in several pathological and non-pathological conditions, including arterial hypertension, pre-eclampsia, male erectile dysfunction, MELAS syndrome and for athletic performance [8,9,10,11,12].

## 2. Results

### 2.1. Osteogenic Function of Arginine In Vitro

**Figure 1:**
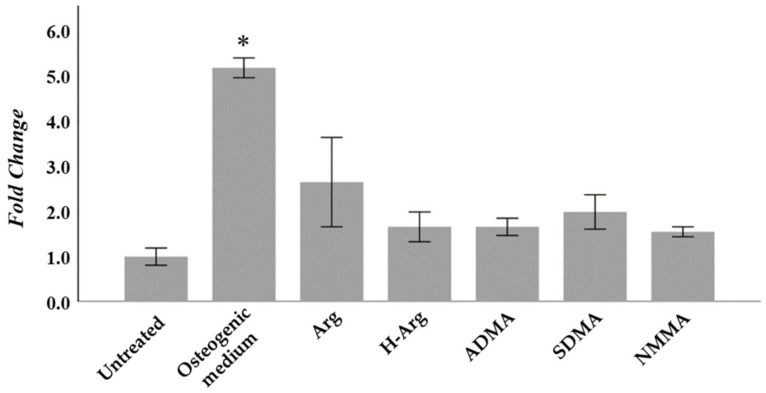
Quantification of calcium deposits by alizarin red staining and spectrophotometric quantitation. Optical density at 405 nm normalized to untreated cells for each treatment after 21 days. Results are the mean ± SD of 3 replicates. * *p* < 0.05 with respect to untreated cells. Arg: L-arginine; H-Arg: L-homoarginine; ADMA: asymmetric dimethyl-L-arginine; SDMA: symmetric dimethyl-L-arginine; NMMA: N^G^-monomethyl-L-arginine.

A quantification of the mineralization process of Saos-2 cells in response to treatments can be seen in Figure 1. As expected, a substantial increase in calcium deposition can be seen in response to osteogenic medium. Treatment with 1 mM arginine also produced a considerable albeit non-significant rise in calcium deposition (fold change: 2.83).

**Figure 2:**
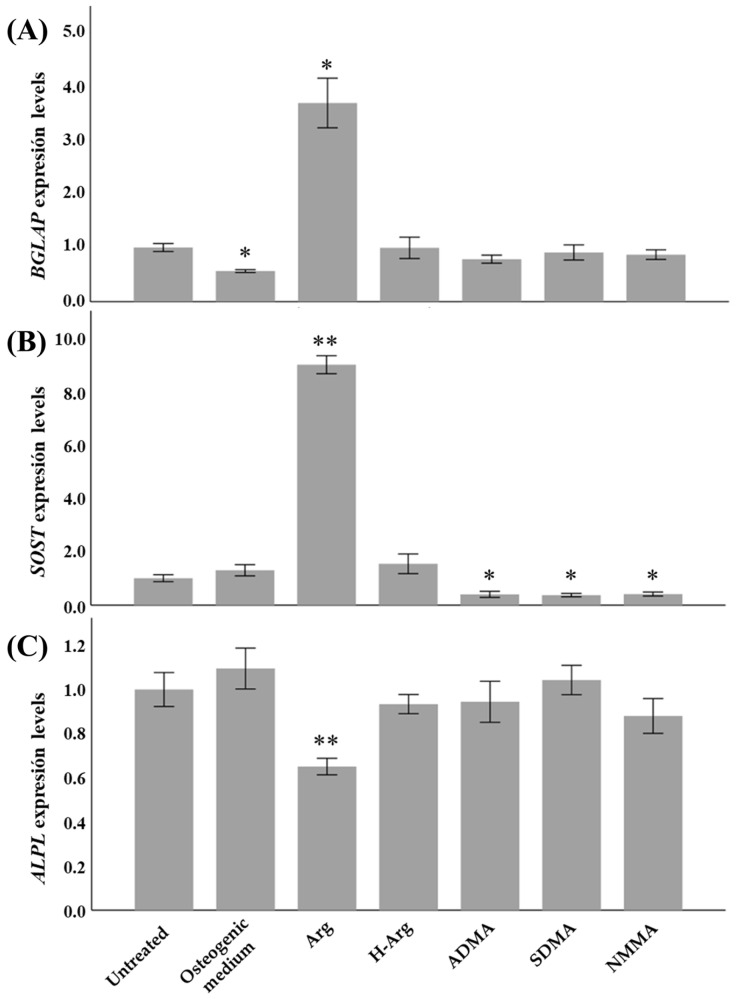
Expression of osteoblastic differentiation marker genes. Expression levels of (**A**) *BGLAP*, (**B**) *SOST* and (**C**) *ALPL* in Saos-2 cells after 21-day treatment. These values were calculated with the 2^−ΔΔCt^. Results are the mean ± SD of 3 replicates. * *p* < 0.05 and ** *p* < 0.001 with respect to untreated cells. Arg: L-arginine; H-Arg: L-homoarginine; ADMA: asymmetric dimethyl-L-arginine; SDMA: symmetric dimethyl-L-arginine; NMMA: N^G^-monomethyl-L-arginine.

Quantitative real time PCR was used to study the expression of marker genes of osteoblastic differentiation in Saos-2 cells (results shown in Figure 2).

*BGLAP* and *SOST*, markers of osteocyte differentiation, showed significantly increased expression (fold change: 3.7 and 8.9 respectively) in response to 1 mM arginine, whereas *SOST* expression was significantly decreased in response to ADMA, SDMA and NMMA. Expression of *ALPL*, considered a marker of osteogenic activity, was slightly but significantly reduced in response to arginine treatment.

### 2.2. Osteogenic Function of Arginine In Vivo

The potential role of arginine supplementation in bone loss was studied in 26 15-week-old female mice divided into four groups: SHAM (n =5), SHAM with treatment (n = 5), OVX (n = 8) and OVX with treatment (n = 8). During the 6-week treatment period, the groups receiving treatment were supplemented with 1% (*w*/*v*) arginine in drinking water. The average water intake of each mouse in the SHAM with treatment group was 4.077 mL per day, resulting in a supplementary intake of 40 mg of arginine daily. Meanwhile, mice in the OVX with treatment group had an average daily water intake of 3.185 mL, constituting a 31 mg daily arginine intake. These approximate values were calculated by subtracting the water volume remaining in the bottle with from the initial volume when it was changed every 3 days.

One mouse from the SHAM with treatment group was eliminated due to reaching its humane endpoint.

After sacrifice, uteri were extracted, cleaned from fat tissue and weighed to verify the success of the ovariectomy. The average uterus weight was significantly (*p* = 0.0006) lower in the OVX mice than in the SHAM mice.

Almost all of the biochemical and hematologic parameters studied (Table 1) were within normal levels except for leucocytes, which were slightly above the range (1.8–5.2 × 10^9^/L) found in the OVX groups (*p* = 0.018), an expected result after the operation [13,14]. This suggests that neither treatment nor ovariectomy had a significant impact on the overall health of the mice. The only parameter showing differences in treatment response was total bilirubin, which exhibited a slight increase (*p* = 0.045). Moreover, weight was not a significant covariate, except for the alanine transaminase (*p* < 0.05) and platelet (*p* < 0.01) variables.

**Figure 3:**
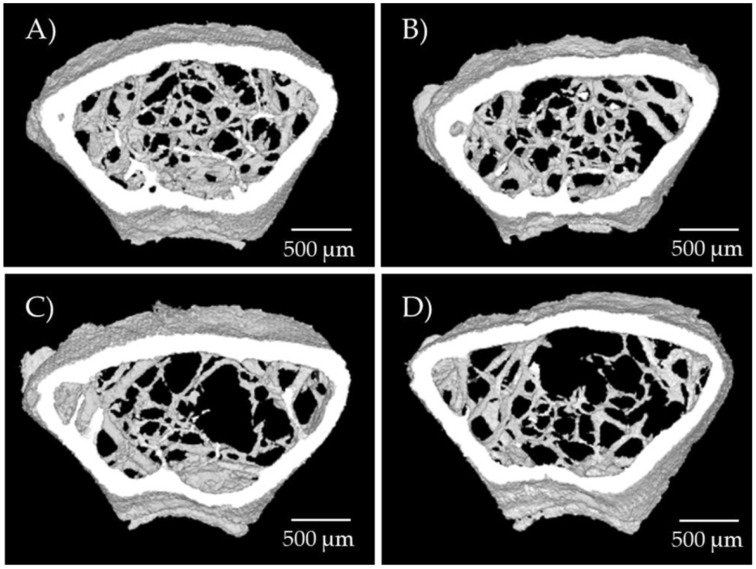
Representative 3D trabecular microarchitectural images of distal mouse femora by means of micro-CT. (**A**) SHAM; (**B**) SHAM with treatment; (**C**) OVX; and (**D**) OVX with treatment.

Table 2 presents the most characteristic parameters of femur microarchitecture obtained with X-ray microtomography for trabecular and cortical bone, while Figure 3 shows representative images of femur bone microarchitecture for each group.

Trabecular BMD (tbBMD), measured in the femoral neck, was lower in the OVX than the SHAM mice, and was also lower in mice with arginine treatment than in those without. The linear univariate model showed a significant fall in tbBMD associated with both OVX (*p* = 0.013) and treatment (*p* = 0.013). The same pattern was observed in BV/TV (probably the parameter that best informs about trabecular bone status), which showed a decrease in response to OVX (*p* = 0.001) and to treatment (*p* = 0.009).

Trabecular thickness was significantly reduced in the OVX groups (*p* = 0.007) and showed a non-significant decrease in the treatment groups (*p* = 0.071). Trabecular number was reduced in response to both OVX (*p* = 0.028) and treatment (*p* = 0.044). As expected, trabecular separation increased significantly in response to OVX (*p* < 0.000). The trabecular bone pattern factor showed a significant treatment-based increase (*p* = 0.001), indicating a more disconnected trabecular structure.

Conversely, cortical bone was exclusively impacted by OVX, with no effect observed from treatment. With the exception of coBMD, therefore, all the variables analyzed exhibited worse results following ovariectomy, particularly CS/BV (*p* < 0.000), which indicates a greater exposed surface area in relation to bone volume, and cortical bone thickness, which measures the thickness of the cortical layer (*p* < 0.000). We also observed a significant decrease in polar moment of inertia (a measure of resistance to rotation forces) in response to OVX (*p* = 0.04), although the *p*-value of the model was not significant (*p* = 0.098).

Regarding analysis of serum biochemical markers of bone remodeling (Table 3), CTx showed a significant increase in response to OVX (*p* = 0.007) and a significant decrease in response to treatment (*p* = 0.041). PINP showed a clear rising trend in response to OVX (*p* = 0.058).

Finally, no significant changes were observed in *Alpl*, *Bglap* and *Sost* gene expression in mouse bone marrow in response to treatment, ovariectomy or to the interaction of the two.

## 3. Discussion

In the present study, we found contrasting effects for arginine, which enhanced osteoblastic differentiation and calcium deposition in vitro, but worsened trabecular bone parameters in vivo. The osteogenic potential of arginine was first studied in vitro by quantifying matrix deposition via the Saos-2 cell line. After a 21-day arginine treatment, we observed an increase in calcium deposition and a parallel increase in the expression of the *BGLAP* and *SOST* genes (markers of osteoblastic differentiation). Bone mineralization is intimately related to osteoblastic differentiation, since OBs are prime secretors of type I collagen and osteocalcin, the most abundant proteins in the bone matrix [15]. The *BGLAP* gene encodes osteocalcin, a protein which promotes osteoid mineralization [16], while the *SOST* gene encodes sclerostin, which is synthetized by osteocytes and inhibits osteoblastogenesis, regulating bone formation [17].

The *ALPL* gene, which encodes alkaline phosphatase, showed a small but significant arginine-induced decrease in expression. Alkaline phosphatase is considered a canonical marker of osteogenic activity; it functions by promoting the availability of inorganic phosphate. Our finding of reduced expression in cells which—according to other parameters—are undergoing osteoblastic differentiation therefore seems contradictory. Nevertheless, mRNA levels may not be an accurate reflection of protein levels.

Taken together, our in vitro results show that L-arginine enhances osteoblastic differentiation, suggesting a potential protective effect on bone loss. In addition to its role in osteoblastic differentiation, arginine could potentially exert indirect effects on osteoclastogenesis by influencing the RANKL/OPG balance, possibly via modulation of SOST expression in osteocytes [18].

Arginine’s in vivo osteogenic potential has been studied in ovariectomized c57BL/6 mice, a model for estrogen depletion-dependent bone loss [19]. The uterine atrophy observed in OVX mice confirms their validity as menopausal models. OVX mice showed a significant decrease in trabecular bone parameters, and to a lesser extent in cortical bone volume and thickness. These results further support that bone loss is due to estrogen depletion, since it causes mainly trabecular bone loss [20,21].

Surprisingly, the arginine-treated groups also exhibited deteriorating trabecular bone parameters, including a notable decrease in femoral neck BMD, BV/TV, trabecular number and connectivity in response to treatment. Conversely, we did not observe significant treatment-related changes in cortical bone parameters.

Analysis of osteogenesis marker gene expression in bone marrow showed no differences in treatment response, contrary to the results obtained in vitro. Although bone marrow contains mesenchymal stem cells rather than osteoblasts, other studies have nonetheless found increased expression of genes implicated in osteoblastic differentiation in mesenchymal stem cells in response to osteogenesis inductors [22].

With respect to serum biochemical markers of bone metabolism, we observed a significant increase in CTx and an increase approaching significance in PINP in response to ovariectomy. CTx is a product of collagen degradation and a widely recognized marker of bone resorption [23], while PINP is split during collagen I formation and its serum levels have been directly correlated with bone matrix formation [24]. The augmented levels of CTx and PINP after OVX reflect an increase in bone turnover that has been widely reported in previous studies [25,26]. In response to treatment, we observed a significant decrease in CTx but not PINP, indicating reduced bone resorption. In a context where arginine has caused bone loss (Table 2), this treatment-induced drop in CTx is unexpected. One possible explanation is that arginine induces bone loss by inhibiting bone formation. While not statistically significant (*p* = 0.192) in the linear univariate model, untreated mice had higher estimated PINP levels (mean ± SE = 73.0 ± 5.3) than treated mice (62.9 ± 5.6), which could support this hypothesis.

These findings point to a detrimental role of arginine in bone metabolism in vivo, which is in contrast with our results obtained in vitro and with other in vivo studies. Treatment with intraperitoneal arginine was described by Pennisi et al. as preventing bone loss associated with type I diabetes in rats after 6 weeks, while treatment with N^G^-nitro-L-arginine methyl ester, a NOS enzyme inhibitor, caused greater BMD loss [27]. While this could be merely counteracting the effect of type I diabetes, Choi et al. described an increase in BMD and mineral content in rat femurs after a nine-week dietary arginine supplementation [28].

The utility of arginine supplementation for wound healing, vascular health and exercise has been suggested in multiple studies for its potential as a NO precursor [29,30,31]. The role of nitric oxide in bone resorption prevention has been widely studied [32], and it has been reported that therapy with nitric oxide donors prevents OVX-related bone loss in rats [33]. Other studies have suggested a protective role for arginine against oxidative stress as an inhibitor and neutralizer of free radicals [34,35]. It has been demonstrated that oxidative stress contributes to bone loss after menopause [36], and antioxidant therapy has been effective in bone loss prevention in OVX rats [37].

Nonetheless, studies in humans on supplementation with arginine or NO precursors have produced heterogeneous results. Baecker et al. reported that 6-month oral arginine supplementation in a cohort of 30 postmenopausal women produced an increase in the serum bone formation marker PICP (Procollagen Type I Carboxy-Terminal Propeptide) but not in BMD [7]. Jamal et al. described an association between nitrate intake and higher BMD in a cohort of 6201 women [38], and it has also been associated with a lower risk of bone fracture [39].

In contrast with previously published results, we observed an effect of arginine on BMD that is not beneficial or neutral but detrimental. This can be ascribed to a systemic detrimental effect of arginine, given that excess administration of intraperitoneal arginine causes pancreatic acinar cell degeneration, serving as a model for pancreatitis [40,41], and chronic pancreatitis is considered a risk factor for the development of secondary OTP [42,43]. However, these effects have not been reported with oral administration, and in our experiment amylase blood levels, a primary indicator of pancreatitis [42], did not show changes in response to treatment. Furthermore, arginine levels were below the maximum levels administered without the negative effects in other studies that used this animal model [44,45].

This detrimental effect of arginine could be mediated by NO, given that a high NO concentration has been found to inhibit OB proliferation and stimulates OC mediated bone resorption [32], which is supported by the fall in tbBMD observed in response to treatment. In any case, interpreting the effects on bone is challenging, especially when different animal models or compounds with distinct mechanisms of action are used. Many beneficial effects attributed to arginine or NO donors have been reported in growing rats [6,28,33], where bone modeling predominates. In contrast, our study used adult mice with a mature skeleton, where bone remodeling is the dominant process. Moreover, the routes of administration, as well as the bioavailability and pharmacokinetics, differ substantially between arginine and organic nitrates. While both can act as NO donors, arginine requires enzymatic conversion by nitric oxide synthase, whereas nitrates are metabolized through alternative enzymatic systems. Additionally, arginine may also give rise to other bioactive metabolites with potential skeletal effects, such as creatine, agmatine, polyamines or ornithine, highlighting the importance of interpreting its effects in the context of the specific experimental model used.

A limitation of the present study is that no histomorphometric analysis of the bone was performed, which would have permitted a more detailed evaluation of bone remodeling dynamics and confirmed the structural findings observed by micro-CT. However, this limitation is partially addressed by the evaluation of circulating bone remodeling markers, which provide complementary information on bone turnover.

In conclusion, our study has yielded contrasting findings on the effects of arginine which should prompt further, wider studies in other populations. Nutritional supplementation (oral administration of nutrients as dietary supplements) has increased steadily over the last two decades [46]. L-arginine is a conditionally essential amino acid whose endogenous synthesis is insufficient in high demand situations. Currently L-arginine-HCl is sold over the counter, commercialized as a dietary supplement in several pathological and non-pathological conditions by many brands [8,9,10,11,12]. Even though arginine supplementation has been proven safe in healthy individuals [47], studies like ours show the necessity of verifying its safety in high doses in other populations or in the presence of pathological conditions.

## 4. Materials and Methods

### 4.1. Study of the Osteogenic Function of Arginine In Vitro

Human osteoblastic Saos-2 cells (ATCC, Manassas, VA, USA) were cultured in 24-well plates using McCoy’s 5a Modified Medium supplemented with 15% fetal bovine serum (FBS, ThermoFisher, Waltham, MA, USA) and 1% penicillin and streptomycin (ThermoFisher) in humidified air containing 5% CO_2_ at 37 °C. The following treatments were added to this medium: osteogenic medium, containing 50 μM L-ascorbic acid and 10 mM beta-glycerophosphate (Merck, Darmstadt, Germany); 1 mM L-Arginine (Arg); 30 μM L-homoarginine (H-Arg); 7 μM asymmetric dimethyl-L-arginine (ADMA); 7 μM symmetric dimethyl-L-arginine (SDMA); and 5 μM NG-monomethyl-L-arginine (NMMA), all from Merck. The added concentrations of the arginine-derived compounds are within the normal range in human serum. Cells were kept for 21 days, medium and treatments were changed every 3 days.

Mineralization was assessed by quantifying calcium deposition using Alizarin Red (Merck) and evaluating the expression of osteoblastic differentiation marker genes.

### 4.2. Quantification of Calcium Mineral Deposition

Culture medium was removed, and adherent cells were washed with PBS and fixed with 4% paraformaldehyde for 15 min, followed by staining with 40 mM Alizarin Red Solution (Merck) for 30 min. After staining, wells were washed abundantly with deionized water and left to dry before storing at −20 °C until dye elution. Mineral-bound dye was rinsed with 10% (*v*/*v*) acetic acid following the protocol previously described [48]. Acid was neutralized with 10% (*v*/*v*) ammonium hydroxide and the absorbance at 405 nm was measured on a VICTOR X Multilabel Plate Reader (Perkin Elmer, Waltham, MA, USA).

### 4.3. Gene Expression

Total RNA was extracted from cells using TRIzol reagent (ThermoFisher) following the manufacturer’s instructions. Retrotranscription of 200 ng of RNA was carried out using the Maxima H Minus First Strand cDNA Synthesis Kit (ThermoFisher) following the manufacturer’s instructions, with both random hexamer primers and oligo(dT). The following reaction cycle: 25 °C for 10 min, 50 °C for 30 min and 85 °C for 5 min was performed in an Applied Biosystems 2720 Thermal Cycler (Applied Biosystems, Foster City, CA, USA).

Real time quantitative amplification of target genes (*ALPL*, *BGLAP*, and *SOST*) and *ACTB* and *GAPDH* as housekeeping was performed using TaqMan Fast Advanced Master Mix (Applied Biosystems), with the specific probe and primers for each gene from the TaqMan Gene Expression Assay kit (ThermoFisher). The assay IDs were: *GAPDH* (Hs02758991_g1), *ACTB* (Hs99999903_m1), *ALPL* (Hs00758162_m1), *BGLAP* (Hs01587814_g1) and *SOST* (Hs00228830_m1). The reaction was carried out in a QuantStudio 5 Real Time PCR System (ThermoFisher) in 384-well plates, and amplification curves were analyzed with QuantStudio Design and Analysis Software (v1.5.1; ThermoFisher).

Normalized values were calculated using the 2^−ΔΔCt^ formula, with ΔCt representing the difference between the target Ct and the mean Ct value of the two housekeeping genes.

### 4.4. Study of Osteogenic Function of Arginine In Vivo

The effect of arginine on bone metabolism and bone mineral density was determined in skeletally mature C57BL/6 female mice (Charles River Laboratories, Barcelona, Spain). The 15-week-old mice were housed upon arrival in an environmentally controlled laboratory at 21 °C, with 12:12 h light–dark, and free access to standard laboratory mouse pellets (containing 0.7% calcium, 0.6% phosphorus and 0.6 IU/g D3 vitamin) and water. After a 3-day acclimatization period, 16 mice were dorsal ovariectomized (OVX) and 10 were falsely operated (SHAM) under general anesthesia using 0.1 mg/kg Butorphanol and 0.3 mg/kg Meloxicam as pre-anesthetic, 5% isoflurane to induce anesthesia and 2% isoflurane to maintain it. The mice were divided into four groups: SHAM (n = 5), SHAM with treatment (n = 5), OVX (n = 8) and OVX with treatment (n = 8). Groups with treatment received 1% (*w*/*v*) arginine with drinking water for 6 weeks. All procedures were reviewed and approved by the research ethics committee of our institution.

All of the mice were sacrificed at 6 weeks after treatment initiation by cardiac puncture exsanguination under halothane anesthesia. The uterus, both femurs and the spleen were aseptically removed. Uteri were weighed to confirm successful ovariectomy. Right femurs were cleaned of adherent soft tissues and stored at −20 °C until analysis. Bone marrow cells were isolated from left femurs by centrifugation as described in [49] and immediately resuspended in TRIzol reagent (ThermoFisher).

Biochemical and hematologic parameters were determined in total blood using the Skyla VB1 analyzer (CVM Diagnóstico Veterinario) and Element HT5 (Heska Corporation, Loveland, CO, USA), respectively.

Serum was obtained by centrifugation at 900× *g* and was employed to determine bone metabolism biomarkers using ELISA. Levels of carboxy-terminal telopeptide of type I collagen (CTx) were detected using the Rat-Laps^®^ (CTX-I) EIA kit (Immunodiagnostic Systems, East Boldon, UK), while procollagen type I N-terminal propeptide (PINP) levels were determined with the Rat/Mouse PINP EIA kit (Immunodiagnostic Systems) following the manufacturer’s instructions.

Total bone marrow RNA was purified using TRIzol Reagent (ThermoFisher) as previously described [50], while RNA concentration and integrity was assessed using the 2100 Bioanalyzer (Agilent Technologies, Santa Clara, CA, USA). For each sample, 200 ng RNA were retrotranscribed using the Maxima H Minus First Strand cDNA Synthesis Kit (ThermoFisher) following the manufacturer’s instructions. Random hexamer primers and oligo(dT) were used, and the reaction was performed in an Applied Biosystems 2720 Thermal Cycler (Applied Biosystems).

Quantitative amplification of *Alpl*, *Bglap* and *Sost* as markers of osteoblastic differentiation, and *Actb* and *Gapdh* as housekeeping genes was performed using TaqMan Fast Advanced Master Mix (Applied Biosystems) and the specific primers and probe contained in the TaqMan Gene Expression Assay kit (ThermoFisher). The assay IDs were: *Gapdh* (Mm99999915_g1), *Actb* (Mm00607939_s1), *Alpl* (Mm01187117_m1), *Bglap* (Mm00649782_gH), and *Sost* (Mm00470479_m1). One μL of the retrotranscription product was amplified in a final volume of 10 μL, and the reaction was performed in the QuantStudio 5 Real Time PCR System (ThermoFisher).

Normalized gene expression values were calculated using the 2^−ΔΔCt^ formula, with ΔCt representing the difference between the target Ct and the mean of the housekeeping genes Cts, to minimize possible gene expression variations.

Three-dimensional microarchitecture of the right femur was analyzed without further preparation by X-ray microtomography in the Bioterio Preclinical Imaging Unit of the University of Oviedo using a SkyScan 1174v2 (Bruker Corporation, Billerica, MA, USA). Images were obtained with an X-ray tube voltage (kV) of 45 and current (mA) of 0.4. The scanning angular rotation was 185°, the angular increment was 0.3° and the resolution was 9.6 μm. Data were reconstructed using a modified Feldkamp algorithm and segmented into 8-bit BMP images [51]. Image analysis was performed with the SkyScan™ CT-analyzer software, version 1.1 (build 10) (Bruker).

To analyze the trabecular bone region, we selected a volume of interest (VOI) between 1.40 and 2.7 mm distance from the proximal growth plate, while cortical bone was analyzed in a VOI spanning from 2.75–3.40 mm from the growth plate. BMD for the cortical and trabecular region was calculated as described in [21].

The main morphometric characteristics of the bone samples were determined from microtomography data using direct 3D morphometry with the marching cubes algorithm. Trabecular bone percentage (BV/TV, %) was calculated from the total volume of the VOI (TV, mm^3^) and the trabecular bone volume (BV, mm^3^) in the VOI. Trabecular thickness (mm), trabecular separation (mm), trabecular number (mm) and trabecular bone pattern factor (1/mm) were measured directly in the 3D images as previously described [52,53]. The morphometric variables measured in the cortical bone region were cortical thickness (mm) and volume (mm^3^), as well as the ratio between cortical surface (mm^2^) and total bone volume (1/mm), and the polar moment of inertia (MMI, mm^4^).

### 4.5. Statistical Analysis

For the in vitro experiments, fixed-effects analysis of variance (ANOVA) was used to compare means between groups, and the Bonferroni test (when the variances were assumed to be equal) or Dunnett’s T3 test (when the variances were assumed to be unequal) were applied to perform post hoc pairwise comparisons at α  =  0.05 level, in which the mean of every group was compared to the untreated group. Levene’s test was used to determine the homogeneity of variance for each dependent variable.

For in vivo experiments, a two-tailed T test was performed to ascertain the difference in uterus mass between the OVX and SHAM mice. For the bone, serum and gene expression parameters studied, quantitative variables were winsorized by replacing outliers (detected by Tukey’s test) with the next highest or lowest non-outlier value, to minimize the influence of outliers. Untransformed data were analyzed for normality using the Kolmogorov–Smirnov test. Variables with non-normal distribution were transformed using rank based inverse transformation and re-analyzed for normality. Mean between-group differences were analysed using a linear univariate model with weight as a covariable using the Statistical Package for Social Sciences (SPSS Inc., Chicago, IL, USA), v. 28.0 for Windows. To facilitate interpretation, all results are presented as untransformed mean ± standard deviation (SD).

Values shown in the text, tables and figures are mean ± SD and percentages, unless otherwise specified. All analyses were two-tailed, and significance was defined as *p* < 0.05.

## 5. Conclusions

In conclusion, our study has produced conflicting results regarding the effects of arginine, which should encourage further, larger-scale research into the effects and safety of administering high doses of arginine in other conditions and in the presence of pathological conditions.

## Figures and Tables

**Table 1:** Biochemical and hematologic parameters. (a) *p* < 0.05 in response to treatment; (b) *p* < 0.05 in response to ovariectomy.

	SHAM (N)	SHAM + Arg (N)	OVX (N)	OVX + Arg (N)	*p*-Value
Biochemical Parameters					
Urea (mg/dL)	41.0 ± 8.2 (5)	40.3 ± 4.4 (4)	41.8 ± 5.7 (8)	45.0 ± 10.8 (7)	0.430
Albumin (g/dL)	4.0 ± 0.3 (5)	3.8 ± 0.6 (4)	3.9 ± 0.2 (8)	3.9 ± 0.1 (7)	0.279
Alkaline phosphatase (U/L)	115.2 ± 6.9 (5)	110.8 ± 30.6 (4)	110.6 ± 7.5 (8)	107.4 ± 5.8 (7)	0.150
Alanine transaminase (U/L)	55.8 ± 52.3 (5)	33.0 ± 5.2 (4)	60.4 ± 38.8 (8)	53.7 ± 50.6 (7)	0.052
Amylase (U/dL)	361.8 ± 133.9 (5)	256.5 ± 47.9 (4)	370.4 ± 283.1 (8)	272.9 ± 32.4 (7)	0.441
Aspartate transaminase (U/dL)	118.4 ± 100.0 (5)	81.5 ± 14.4 (4)	119.0 ± 55.3 (8)	97.3 ± 50.9 (7)	0.309
Blood urea nitrogen(mg/dL)	19.1 ± 3.8 (5)	18.9 ± 2.1 (4)	19.5 ± 2.7 (8)	21.0 ± 5.0 (7)	0.430
Cholesterol (mg/dL)	91.8 ± 10.8 (5)	91.5 ± 3.1 (4)	99.0 ± 6.3 (8)	93.1 ± 17.2 (7)	0.428
Creatinine (mg/dL)	0.38 ± 0.05 (5)	0.35 ± 0.06 (4)	0.34 ± 0.08 (8)	0.38 ± 0.04 (7)	0.202
Calcium (mg/dL)	9.9 ± 0.6 (5)	9.8 ± 0.4 (4)	9.9 ± 0.4 (8)	9.8 ± 0.2 (7)	0.521
Glucose (mg/dL)	224.6 ± 38.2 (5)	203.0 ± 38.7 (4)	206.8 ± 23.9 (8)	201.1 ± 46.0 (7)	0.205
Phosphate (mg/dL)	6.6 ± 1.0 (5)	6.8 ± 1.9 (4)	6.9 ± 2.1 (8)	5.7 ± 1.0 (7)	0.326
Total bilirubin (mg/dL)	0.32 ± 0.11 (5)	0.35 ± 0.27 (4)	0.21 ± 0.18 (8)	0.44 ± 0.19 (7)	0.045 (a)
Total protein (mg/dL)	5.1 ± 0.4 (5)	5.4 ± 0.4 (4)	5.3 ± 0.3 (8)	5.1 ± 0.2 (7)	0.619
Hematopoietic parameters					
Hemoglobin (g/dL)	14.8 ± 0.4 (5)	14.2 ± 1.2 (4)	14.4 ± 0.5 (8)	14.3 ± 0.9 (8)	0.420
Haematocrit (%)	44.1 ± 1.0 (5)	42.5 ± 3.4 (4)	42.9 ± 1.2 (8)	43.0 ± 2.1 (8)	0.488
Erythrocytes (10^12^/L)	9.5 ± 0.1 (5)	9.3 ± 0.6 (4)	9.3 ± 0.3 (8)	9.2 ± 0.6 (8)	0.439
Leukocytes (10^9^/L)	4.8 ± 2.0 (5)	3.9 ± 1.0 (4)	7.1 ± 4.7 (8)	6.8 ± 1.8 (8)	0.018 (b)
Platelets (10^9^/L)	584.2 ± 330.1 (5)	487.5 ± 377.2 (4)	750.4 ± 401.4 (8)	797.0 ± 404.6 (8)	0.054

SHAM: falsely operated; OVX: ovariectomized; Arg: L-arginine.

**Table 2:** Femur microarchitecture parameters obtained using X-ray microtomography after 6-week arginine treatment. (a) *p* < 0.05 in response to ovariectomy; (b) *p* < 0.05 in response to treatment; (c) *p* < 0.01 in response to ovariectomy; (d) *p* < 0.01 in response to treatment; (e) *p* < 0.000 in response to ovariectomy.

	SHAM (N)	SHAM + Arg (N)	OVX (N)	OVX + Arg (N)	*p*-Value
tbBMD (g/cm^3^)	0.132 ± 0.021 (4)	0.120 ± 0.010 (3)	0.117 ± 0.011 (7)	0.095 ± 0.018 (8)	0.002 (a, b)
BV/TV (%)	6.3 ± 0.5 (4)	5.6 ± 0.4 (3)	5.3 ± 0.8 (7)	4.5 ± 0.5 (8)	<0.001 (c, d)
Trabecular thickness (mm)	0.045 ± 0.004 (4)	0.042 ± 0.001 (3)	0.042 ± 0.002 (7)	0.040 ± 0.001 (8)	0.005 (c)
Number of trabeculae (1/mm)	1.36 ± 0.10 (4)	1.33 ± 0.07 (3)	1.28 ± 0.17 (7)	1.12 ± 0.11 (8)	0.011 (a, b)
Trabecular separation (mm)	0.278 ± 0.007 (4)	0.278 ± 0.009 (3)	0.310 ± 0.020 (7)	0.315 ± 0.025 (8)	<0.001 (e)
Trabecular bone pattern factor (1/mm)	35.2 ± 1.7 (4)	38.7 ± 0.6 (3)	36.4 ± 1.9 (7)	38.8 ± 2.0 (8)	0.002 (d)
coBMD (g/cm^3^)	1.27 ± 0.02 (5)	1.27 ± 0.01 (4)	1.26 ± 0.02 (8)	1.27 ± 0.02 (8)	0.285
Cortical bone volume (mm^3^)	0.663 ± 0.040 (5)	0.693 ± 0.057 (4)	0.625 ± 0.048 (8)	0.610 ± 0.023 (8)	0.015 (a)
CS/BV (%)	15.2 ± 0.3 (5)	15.1 ± 0.1 (4)	16.8 ± 0.5 (8)	16.7 ± 0.3 (8)	<0.001 (e)
Cortical bone thickness (mm)	0.183 ± 0.008 (5)	0.185 ± 0.002 (4)	0.170 ± 0.005 (8)	0.167 ± 0.004 (8)	<0.001 (e)
Polar moment of inertia (mm^4^)	0.339 ± 0.044 (5)	0.362 ± 0.063 (4)	0.319 ± 0.017 (8)	0.314 ± 0.024 (8)	0.098 (a)

SHAM: falsely operated; OVX: ovariectomized; Arg: L-arginine; tbBMD: trabecular bone mineral density; coBMD: cortical bone mineral density; BV/TV: trabecular bone percentage; CS/BV: cortical surface/total bone volume. Inconsistencies in sample size between the parameters of the same group are due to femur breakage during the extraction process precluding the measurement of several bones.

**Table 3:** Levels of biochemical markers of bone remodeling in mice serum after 6-week treatment. (a) *p* < 0.01 in response to ovariectomy; (b) *p* < 0.05 in response to treatment (c) *p* < 0.1 in response to ovariectomy.

Biochemical Markers	SHAM (N)	SHAM + Arg (N)	OVX (N)	OVX + Arg (N)	*p*-Value
CTx (ng/mL)	28.4 ± 23.3 (5)	9.6 ± 0.9 (4)	26.6 ± 15.0 (8)	21.5 ± 8.5 (8)	0.005 (a, b)
PINP (ng/mL)	67.6 ± 23.8 (5)	51.1 ± 24.8 (4)	79.4 ± 18.7 (8)	72.9 ± 12.1 (8)	0.058 (c)
ALP (units/mL)	131.3 ± 15.1 (5)	128.5 ± 61.7 (4)	128.2 ± 17.1 (8)	122.6 ± 15.1 (8)	0.127

SHAM: falsely operated; OVX: ovariectomized; Arg: L-arginine; CTx: carboxy-terminal telopeptide of type I collagen; PINP: procollagen type I N-terminal propeptide; ALP: alkaline phosphatase; ALP unit: one unit will hydrolyze 1 μmole of *p*-nitrophenyl phosphate per minute at pH 9.8 at 37 °C.

## Data Availability

Data is contained within the article.
